# Multifaced Assessment of Antioxidant Power, Phytochemical Metabolomics, In-Vitro Biological Potential and In-Silico Studies of *Neurada procumbens* L.: An Important Medicinal Plant

**DOI:** 10.3390/molecules27185849

**Published:** 2022-09-09

**Authors:** Umair Khurshid, Saeed Ahmad, Hammad Saleem, Arslan Hussain LodhI, Irfan Pervaiz, Mohsin Abbas Khan, Haroon Khan, Abdulwahab AlamrI, Mukhtar AnsarI, Marcello LocatellI, Muhammad Adeel Arshad, Muhammad Asif Wazir, Juwairiya Butt, Sirajudheen Anwar

**Affiliations:** 1 Department of Pharmaceutical Chemistry, Faculty of Pharmacy, The Islamia University of Bahawalpur, Bahawalpur 63100, Pakistan; 2 Institute of Pharmaceutical Sciences (IPS), University of Veterinary & Animal Sciences (UVAS), Lahore 54000, Pakistan; 3 Department of Pharmacology, Faculty of Pharmacy, The Islamia University of Bahawalpur, Bahawalpur 63100, Pakistan; 4 Department of Pharmacy, University of Chenab, Gujrat 50700, Pakistan; 5 Gomal Centre of Pharmaceutical Sciences Faculty of Pharmacy, Gomal University, Dera Ismail Khan 29050, Pakistan; 6 Department of Pharmacology and Toxicology, College of Pharmacy, University of Hail, Hail 81451, Saudi Arabia; 7 Department of Clinical Pharmacy, College of Pharmacy, University of Hail, Hail 81451, Saudi Arabia; 8 Department of Pharmacy, University ‘G. d’Annunzio” of Chieti-Pescara, 66100 Chieti, Italy; 9 Institute of Pharmacy, Faculty of Pharmaceutical and Allied Health sciences, Lahore College for Women University, Lahore 54000, Pakistan; 10 Department of Pharmacognosy, Faculty of Pharmacy, The Islamia University of Bahawalpur, Bahawalpur 63100, Pakistan; 11 School of Life Sciences, University of Westminster, 115 New Cavendish Street, London W1W 6UW, UK

**Keywords:** *Neurada procumbens*, HPLC-PDA, GC-MS, secondary metabolites, antioxidant, enzyme inhibition, docking

## Abstract

This work was undertaken to explore the phytochemical composition, antioxidant, and enzyme-inhibiting properties of *Neurada procumbens* L. extracts/fractions of varying polarity (methanol extract and its fractions including *n*-hexane, chloroform, *n*-butanol, and aqueous fractions). A preliminary phytochemical study of all extracts/fractions, HPLC-PDA polyphenolic quantification, and GC-MS analysis of the *n*-hexane fraction were used to identify the phytochemical makeup. Antioxidant (DPPH), enzyme inhibition (against xanthine oxidase, carbonic anhydrase, and urease enzymes), and antibacterial activities against seven bacterial strains were performed for biological investigation. The GC-MS analysis revealed the tentative identification of 22 distinct phytochemicals in the *n*-hexane fraction, the majority of which belonged to the phenol, flavonoid, sesquiterpenoid, terpene, fatty acid, sterol, and triterpenoid classes of secondary metabolites. HPLC-PDA analysis quantified syringic acid, 3-OH benzoic acid, *t*-ferullic acid, naringin, and epicatechin in a significant amount. All of the studied extracts/fractions displayed significant antioxidant capability, with methanol extract exhibiting the highest radical-scavenging activity, as measured by an inhibitory percentage of 81.4 ± 0.7 and an IC_50_ value of 1.3 ± 0.3. For enzyme inhibition experiments, the *n*-hexane fraction was shown to be highly potent against xanthine oxidase and urease enzymes, with respective IC_50_ values of 2.3 ± 0.5 and 1.1 ± 0.4 mg/mL. Similarly, the methanol extract demonstrated the strongest activity against the carbonic anhydrase enzyme, with an IC_50_ value of 2.2 ± 0.4 mg/mL. Moreover, all the studied extracts/fractions presented moderate antibacterial potential against seven bacterial strains. Molecular docking of the five molecules β-amyrin, campesterol, ergosta-4,6,22-trien-3β-ol, stigmasterol, and caryophyllene revealed the interaction of these ligands with the investigated enzyme (xanthine oxidase). The results of the present study suggested that the *N. procumbens* plant may be evaluated as a possible source of bioactive compounds with multifunctional therapeutic applications.

## 1. Introduction

The value of natural products in the conception of therapeutic moieties has been amply shown, and they continue to be an essential resource for identifying new drug leads [1]. From the perspective of the World Health Organization (WHO), it is a profitable endeavour to promote medicinal plants as the source of products/molecules for both customary and modern medicine [2]. Historically, a bigger proportion of the pharmaceutical business has been dominated by medications derived from natural sources. The exploration of natural compounds as a source of innovative human therapies is of utmost relevance [3]. Due to the vast therapeutic potential of medicinal plants against a variety of ailments, there is a strong connection between them and the invention of new drugs. The traditional use of these herbs is sufficient evidence in this regard [4].

Due to their pharmacological activity (antioxidant, anticancer, antimicrobial, and enzyme inhibiting potential, etc.) as well as their nutritional health advantages, the medicinal potential of plants has achieved worldwide significance with the growth of science. Standard combinatorial chemistry produces less structural variety than natural products, hence plants contribute to the identification of novel lead compounds against many assay targets. It is expected that the presence of a large number of pharmacophores and a high degree of stereochemistry are linked to the ability of natural products to bind to more complex targets, such as proteins. Natural products exhibit structural and chemical differences that are incomparable to small molecular libraries and continue to inspire new discoveries in chemistry, biology, and medicine. The best source of therapeutic leads is still molecules isolated from natural products that have been evolutionarily improved.

Natural products from a variety of sources are again being sought out as a source of pharmaceuticals, despite the fact that the pharmaceutical industry has grown and organic chemistry understanding has increased dramatically, which has led to a preference for synthetic treatments. There are natural antecedents to a range of synthesized drugs, according to the WHO’s estimate that 11% of the 252 approved medications are derived totally from plants [5]. Serpentine from *Rauwolfia* revolutionized the treatment of hypertension and served as a forerunner for the development of synthetic equivalents with greater efficacy and lower toxicity, both of which were developed as a result of plant-derived pharmaceuticals. They found that Catharanthus Vinblastine and Vincristine have good anticancer action. Podophyllotoxin found in *Podophyllum*, is also used to treat lymphomas [6,7,8].

Many therapeutic aims, including Alzheimer’s disease, HIV/AIDS, cancer, malaria and pain, have been met through the use of plant-derived lead compounds. Paclitaxel, camptothecin-derived analogues, artemether, galantamine, tiotropium bromide, and other plant-derived natural medicines are being utilized in clinical trials. Many hurdles have been overcome in the acquisition, certification, execution and expansion of lead compounds in drug discovery from natural origin [9].

The traditional name for this plant is “Chapri-booti”; North Africa and the Middle East to Pakistan and India are home to this annual herb [10]. The plant is used to wilting, sandy conditions, growing up to 1-metre across. Stems with hairy branches lie flat on sand, giving the impression of being fuzzy. Single off-white flowers encased in a ring of bluish-green leaves are common in this species. Smooth on the inside and crisp on the outside, this star-shaped fruit has a smooth inside. With its long history as a sexual stimulant, general tonic, and nerve relaxant, *N. procumbens* is still widely used today [11]. It is also used as an anti-diarrheal and as a cardio-tonic [12]. The anti-viral and anti-cancer effects of this plant have previously been studied [13,14]. Anaesthetized normotensive rats’ blood pressure was found to be increased after oral administration of this plant’s aqueous extract. For cooling in the summer and calming the nerves in the winter, its powdered fruit can be utilized [15,16,17].

It was shown that *Neurada procumbens* has anti-cancer properties. Tests on cancer cell lines from breast (MCF-7), colon (HCT-116) and hepatic cells have shown that the plant’s methanolic extract is effective in killing all these cell lines including lung cancer cells (A-549). The four cancer cell lines listed above showed mild to moderate activity [18]. New Castle Disease Virus (NDV) was successfully treated with the methanolic extract of *Neurada procumbens*. Six flavonoids were previously identified and reported from the plant’s *n*-butanolic fraction aerial parts. These compounds were identified as the following: taxifolin, astilbin (taxifolin 3-*O*-rhamnopyranoside), vitexin, orientin 200-*O*-a-rhamnopyranoside, and isoorientin 200-*O*-a-rhamnopyranoside [12,13,14].

Acknowledging the significance of plants as medicinal products and as a core for lead compounds, it is necessary to conduct appropriate and methodical phytochemical studies. The recent study aspires to study the biologically active natural products of the medicinal plant *Neuroda procumbens* found in the desert areas of Pakistan thereby supporting the orthodox use of plants and providing achievable clues for discovery of novel drugs.

## 2. Results

### 2.1. Phytochemical Analysis

Preliminary qualitative phytochemicals assessment of the methanolic extract, and different fractions (*n*-hexane, chloroform, *n*-butanol, and aqueous fractions) of *N. procumbens* were performed and the results are presented in Table 1. The preliminary phytochemical testing of *N. procumbens* showed that the tested extract/fractions are a rich source of alkaloids, flavonoids, phenols, saponins, glycosides, tannins, steroids, coumarins, quinones and lipids. 

**Figure 1:**
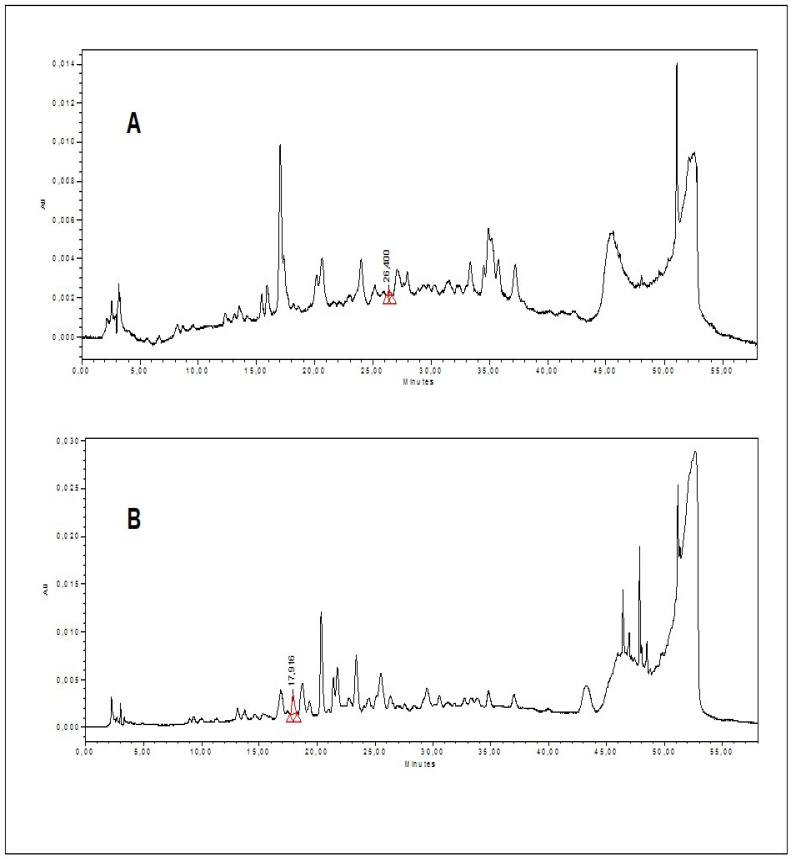

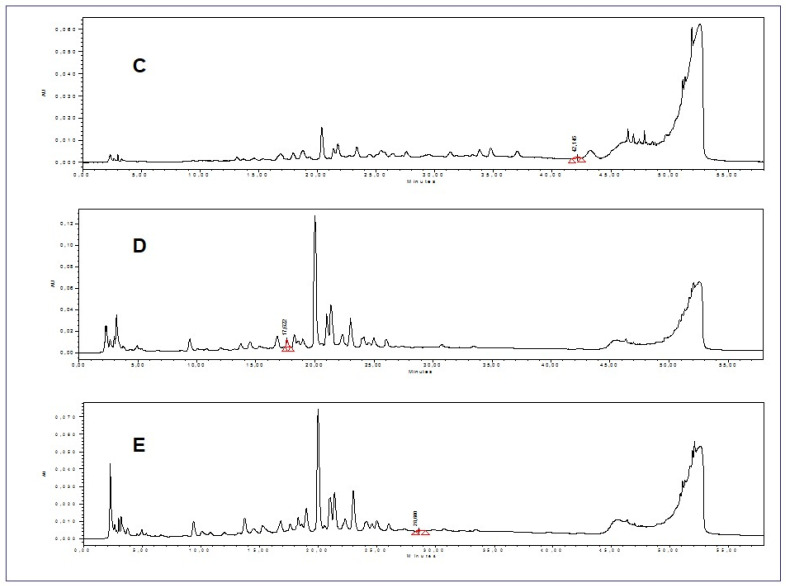
HPLC-PDA chromatograms. (**A**) *t*-ferullic acid, (**B**) 3-OH benzoic acid. (**C**) Syringic acid, (**D**) Naringin, and (**E**) Harpagoside.

Similarly, to gain a more in-depth insight into the phytochemical composition, HPLC-PDA analysis of the studied plant extracts was carried out. A list of 22 important standard phenolic phytochemicals were tested for their quantification in all the extracts/fractions of *N. procumbens.* However, all the studied extracts were found to be quantified for eight of these compounds. The results of these quantified phenolics are presented in Table 2, and their respective HPLC-PDA chromatograms are shown in Figure 1. The presence of 3-OH benzoic acid (1.9 ± 0.2 μg/g), *t*-ferullic acid (0.3 ± 0.02 μg/g), and harpagoside (2.4 ± 0.8 μg/g) was observed in the chloroform extract of *N. procumbens*, followed by the presence of syringic acid (3.6 ± 0.4 μg/g) and naringin (0.5 ± 0.05 μg/g) in the *n*-butanol extract. However, none of the 22 phenolic compounds tested were found in *n*-hexane extract.

**Figure 2:**
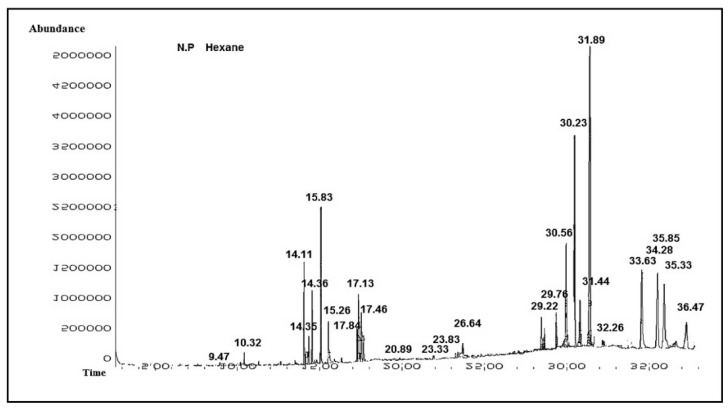
GC-MS chromatogram of *Neurada procumbens n*-hexane fraction.

Furthermore, GC-MS analysis was employed to have detailed individual secondary metabolites profiling. The tentative identification of secondary metabolites from *n*-hexane fraction of *N. procumbens* was carried out using GC-MS analysis. The GC-MS spectra exhibiting altered peaks of the tentatively identified compounds are illustrated in Figure 2. The *n*-hexane fraction unveiled 22 compounds presented in Table 3 which belonged to the alkanes, alkenes, phenol, phytosterol, fatty acid ester, diterpenoid, tocopherol, benzene derivative, and sesquiterpene classes of secondary metabolites.

### 2.2. Antioxidant Assay

Table 4 showed that methanolic extract exhibited the highest activity 81.37 ± 0.72 with IC_50_ value of 1.13 ± 0.3 while chloroform fraction displayed the lowest inhibitory potential 43.47 ± 0.61 with IC_50_ of 6.42 ± 1.8. Antioxidant potential measured through DPPH assay exhibited percentage inhibition in following sequence methanolic > *n*-butanol > *n*-hexane > chloroform > aqueous (81.37 ± 0.72 > 74.22 ± 0.59 > 67.19 ± 0.85 > 43.47 ± 0.61 > 18.84 ± 0.92).

### 2.3. Enzyme Inhibition Assays

The studied plant extracts were tested against different enzymes, including xanthine oxidae, carbonic anhydrase and urease. The standard used for xanthine oxidase was allopurinol and the results were presented in percentage inhibition followed by IC_50_ in mg/mL. Acetazolamide was used as standard for carbonic anhydrase and thio-urea for urease enzyme. The inhibitory potential along with IC_50_ values of plant extract/fractions against all three enzymes is displayed in Table 5. The maximum percentage inhibition against xanthine oxidase was displayed by *n*-hexane fraction 79.5 ± 0.9 (IC_50_ 2.3 ± 0.5 mg/mL), while *n*-butanol fraction exhibited the lowest potential. In the case of carbonic anhydrase enzyme, methanolic extract of *N. procumbens* exhibited the highest activity (IC_50_ 2.2 ± 0.4 mg/mL), followed by the *n*-butanol fraction (IC_50_ 3.1 ± 0.4 mg/mL). The inhibitory potential of the other fractions against carbonic anhydrase was insignificant. The results of the anti-urease assay were supported by the GC-MS analysis of the *n*-hexane fraction with maximum activity (84.7 ± 1.9 IC_50_ 1.1 ± 0.4 mg/mL).

### 2.4. Antibacterial Activity

**Figure 3:**
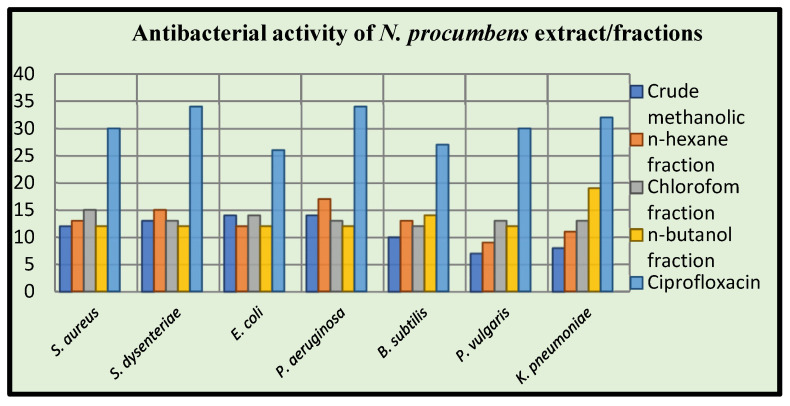
Graphical representation of antibacterial activity of *Neurada procumbens*.

The antibacterial activity was evaluated using agar well diffusion method with ciprofloxacin as standard drug. Seven bacterial strains, including *S. aureus, S. dysenteriae, E. coli, P. aeruginosa, B. subtilis, P. vulgaris*, and *K. pneumonia*, associated with various diseases, were tested in an antibacterial assay using different fractions of *N. procumbens* based on polarity. Table 6 and Figure 3 depicts the results in terms of zones of inhibition. In the present study, methanolic extract of *N. procumbens* exhibited an inhibition zone ranging from 7 to 16 mm against various bacteria, *n*-hexane fraction exhibited an inhibition zone ranging from 9 to 17 mm, chloroform fraction exhibited an inhibition zone ranging from 12 to 19 mm, and *n*-butanol fraction exhibited an inhibition zone ranging from 12 to 19 mm against the tested bacteria. The chloroform fraction showed the highest inhibitory zone of 19 ± 0.1 against *E. coli* and lowest against *B. subtilis* 12 ± 0.6, while *n*-hexane fraction displayed a significant zone of inhibition against *P. aeruginosa* (17 ± 0.6) and the lowest with *P. vulgaris* (9 ± 0.7). An inhibitory zone of 19 ± 0.1 was assessed against *K. pneumoniae* by *n*-butanol fraction and 16 ± 0.8 against *E. coli* by methanolic extract. Aqueous fraction presented insignificant results against all the bacterial strains.

### 2.5. In-Silico Studies

**Figure 4:**
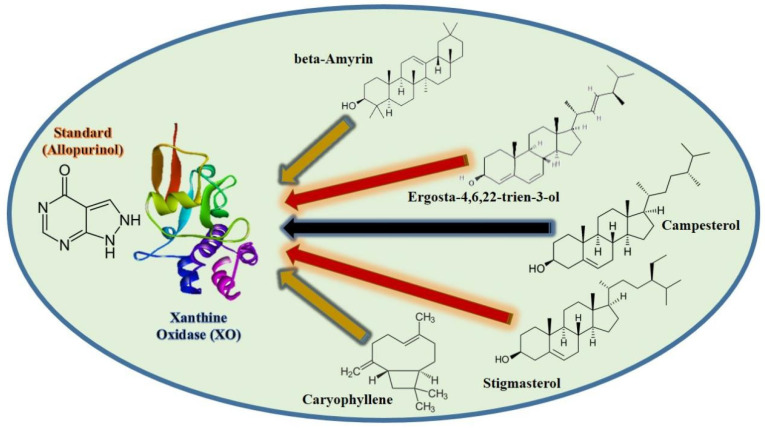
Molecular docking of selected ligands with xanthine oxidase enzyme.

**Figure 5:**
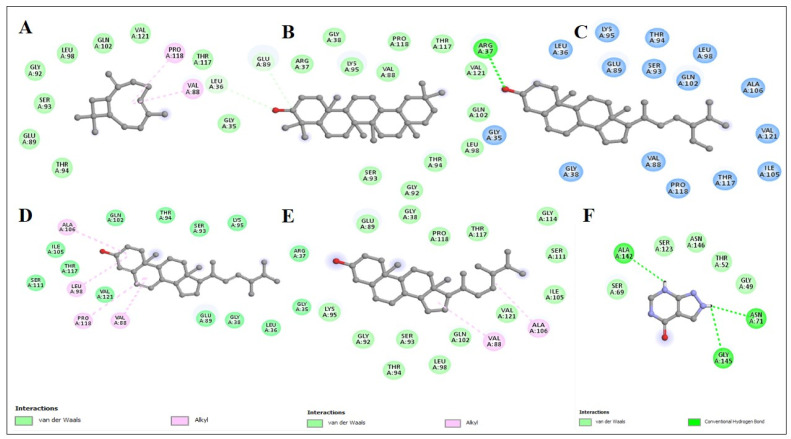
Two-dimensional structured binding interactions of Caryophyllene (**A**), β-Amyrin (**B**), Stigmasterol (**C**), Ergosta-4,6,22-trien-3-ol (**D**), Campesterol (**E**), and Allopurinol (standard) (**F**) with xanthine oxidase enzyme.

**Figure 6:**
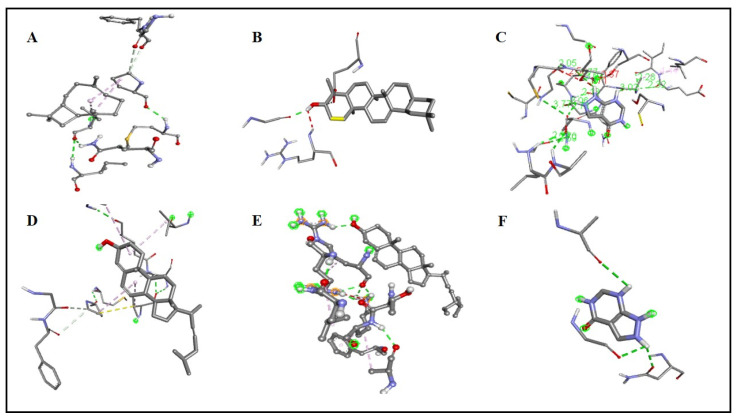
Three-dimensional structured molecular interactions of Caryophyllene (**A**), β-Amyrin (**B**), Stigmasterol (**C**), Ergosta-4,6,22-trien-3-ol (**D**), Campesterol (**E**), and Allopurinol (standard) (**F**) with Xanthine oxidase enzyme.

A total of 26 ligands were docked with the enzymes. Five compounds were chosen based on their high energy affinity shown in Figure 4. Additionally, alkyl, pi alkyl, and conventional hydrogen bond interactions with the amino acids were on the higher side of the binding affinity of β-amyrin (−9.7). Xanthine oxidase amino acids had a lot of interactions with β-amyrin, as did other amino acids close to the enzyme’s active region. That could have a biologically important effect on the local structure. Validation of the molecular docking was conducted using Autodock-1.5.6, which re-docked the enzyme and selected ligands. Binding affinity and root-mean-square deviation were also determined to be identical. Receptor-xanthine oxidase interactions (both 2D and 3D) with ligands are shown in Figure 5 and Figure 6, while the binding affinities and binding forces of the ligands and enzyme are reported in Table 7.

## 3. Discussion

Analysing the phytochemistry of a plant is essential for determining its probable medical uses and for identifying the active components responsible for its recognized biological activity. Additionally, it provides the foundation for the targeted separation of chemicals and more precise research. The phytochemical screening of *N. procumbens* extract/fractions revealed that the plant is ultimate source of tannins, saponins, flavonoids, lipids, alkaloids, and phenols.

According to the scientific reports, glycosides, tannins, alkaloids and resins are involved in possessing anti-bacterial potential [19,20]; polyphenols generally and flavonoids particularly exhibited anti-bacterial and antioxidant potentials [21]; quinones are folklore therapies in reducing mitochondrial oxidative stress [22]; while coumarins have been found to possess antioxidant, anti-microbial and enzyme inhibition properties [23]. The present study confirmed the presence of these phyto-constituents in *N. procumbens* so their presence might contribute to its therapeutic potential.

*N. procumbens* chloroform extract had higher levels of phenolics than the other extracts/fractions, including higher concentrations of 3-OH benzoic acid (1.9 ± 0.2 μg/g extract), *t*-ferullic acid (0.3 ± 0.02 μg/g extract), and harpagoside (2.4 ± 0.8 μg/g extract), while syringic acid (3.6 ± 0.4 μg/g extract) and naringin (0.5 ± 0.05) quantified in *n*-butanol fraction with catechin was noted to be below the detection limit (BLD). The methanolic extract only contained *t*-ferullic acid (1.7 ± 0.6 μg/g extract) in significant quantities. The *n*-hexane fraction did not contain any of the tested phenolic standards in significant amounts with the presence of gallic acid and quercetin but in below the detection limit (BLD). To summarize, the results of this phenolic profiling show that important secondary metabolites are present, allowing further investigation into the isolation of bioactive molecules with potential importance.

The putative identification of secondary metabolites from the *n*-hexane fraction of *N. procumbens* was investigated using GC-MS. The *n*-hexane fraction unearthed 26 secondary metabolites belonging to alkanes, alkenes, phenol, phytosterol, fatty acid ester, diterpenoid, tocopherol, benzene derivative, and sesquiterpene families. The identified compounds included caryophyllene, phenol, 2,5-bis(1,1-dimethyleth, bicyclo[3.1.1]heptane, 2,6,6-tr, 2-pentadecanone, 6,10,14-trimet, 5-nonadecen-1-ol, hexadecanoic acid, methyl ester, *n*-hexadecanoic acid, 9,12,15-octadecatrienoic acid, phytol, octadecanoic acid, eicosanoic acid, methyl ester, docosanoic acid, 1,2-Benzenedicarboxylic acid, tetracosanoic acid, methoxyacetic acid, cholest-5-en-3-ol (3β), stigmastan-3,5,22-trien, (22*E*)-ergosta-4,6,22-trien-3-ol, stigmastan-3,5-diene, 1-(3-hydroxy-4-methylphenyl)-, d,α-tocopherol, campesterol, stigmasterol, octacosane, stigmasterol, and β-amyrin.

Reactive oxygen species are frequently produced during metabolic processes (ROS). Toxic build-up of ROS damages fatty acids, DNA, and proteins, resulting in tissue destruction and inflammation. Organic component DPPH is used to test plant extract’s antioxidant potential. Picrylhydrazine, a pale-yellow radical, is decreased by antioxidant components in plant extracts [24]. The natural anti-oxidant defence system in living beings controls gene mutation and molecular transformation by free radicals. Antioxidants in food can be found in a wide variety of herbs, spices, and plant extracts and products

In the current research there was a significant difference in activity between the highest activity displayed by methanolic extract (IC_50_ 1.1 ± 0.3 mg/mL) and the other two extracts, *n*-butanol (IC_50_ 1.5 ± 0.3mg/mL) and the fraction of the *n*-hexane (IC_50_ 2.2 ± 0.5 mg/mL) presented in Table 3. The DPPH (radical scavenging) activity of all *N. procumbens* fractions except the chloroform fraction was found to be significant.

One of the most important enzymes in the human body is the flavoprotein xanthine oxidase (XO). This enzyme is found throughout the animal kingdom and in all tissues, from bacteria to humans. One of the most common causes of sickness or at least its symptoms is an imbalance of a specific metabolic acid. Gout can be caused by an excess of uric acid, which is a metabolite that is overproduced in the human body. As XO is inhibited, uric acid levels are reduced, which has an anti-hyperuricemic benefit. One of the earliest drugs developed as an anticancer agent, Allopurinol, is currently used to treat Gout [25]. Xanthine oxidase serum levels are dramatically elevated in several pathological states, such as hepatitis, inflammation, ischemia-reperfusion, carcinogenesis, and ageing, and ROS created in the enzymatic process are implicated in oxidative damage. According to these findings, it is feasible that the blockage of this enzyme pathway might be helpful therapeutically [26].

Xanthine oxidase inhibition was assessed in crude and fractionated extracts of *N. procumbens* and *n*-hexane fraction, with an IC_50_ of 2.3 ± 0.5 mg/mL, noted to be most active, followed by methanol extract, with an IC_50_ of 3.9 ± 0.2 mg/mL, and *n*-butanol fraction, with an IC_50_ of 4.1 ± 0.3 mg/mL. *N. procumbens* has shown promising results in the treatment of disorders associated with elevated levels of uric acid.

Cancer, high altitude sickness, obesity, osteoporosis, and high blood pressure are just some of the conditions that carbonic anhydrase (CA) inhibitors research has shown to have a substantial impact on [27]. As a result of high intraocular pressure (IOP), which alters the optic nerve head and results in visual field loss, glaucoma is considered chronic and progressive disease [28,29]. Eventually, the optic nerve is damaged to the point of blindness. Glaucoma is the second leading cause of blindness in the world, affecting an estimated 70 million people [30]. CA isoforms have been identified as a promising and effective target for glaucoma treatment by reducing IOP in recent decades [31].

The maximum activity against carbonic anhydrase was found in the methanolic extract of *N. procumbens* (IC_50_ 2.2 ± 0.4 g/mL), followed closely by the *n*-butanol fraction (IC_50_ 3.1 ± 0.4 g/mL). The carbonic anhydrase inhibitory potential of the other fractions was negligible.

Antiulcer and anti-gastric cancer drugs can benefit greatly from pharmacological inhibition of the metallo-enzyme urease. Infections caused by Helicobacter pylori in the gastrointestinal tract as well as Proteus and similar species in the urinary tract are often induced by urease. These chemicals, which are employed in the treatment of stomach and urinary infections, have all shown serious side effects, toxicity and instability despite several research reporting on new urease inhibitors. As a result, natural compounds must be explored that have less adverse effects, lower toxicity and improved stability and bioavailability to solve these issues. Due to a lack of research on plant-derived UIs, this work seeks to identify and describe the most promising plant compounds, such as terpenoids, phenolic compounds, alkaloid and other substances having inhibitory actions against plant and bacterial ureases [32].

It is possible that sesquiterpene hydrocarbons or alcohols, or a combination of the two, have potent antibacterial effects on bacterial urease. *H. pylori* may be prevented from adhering to the stomach mucosa by urease inhibition. Some flavonoids have been shown to suppress *H. pylori* in prior investigations [33]. Anti-urease assay results are supported by GC-MS analysis of the fraction’s *n*-hexane. This revealed the presence of sesquiterpenoids, diterpenoids, phenols, and a slew of other chemicals. According to the results of this study, the *n*-hexane fraction had a considerable inhibitory potential of 84.7 ± 1.9 with an IC_50_ of 1.1 ± 0.4 mg/mL.

There are numerous illnesses caused by the gram-positive bacteria *S. aureus*. These include urinary tract infections (UTI), gastroenteritis, toxic shock syndrome (TSS), septicaemia, skin infections, pneumonia, bacteraemia, endocarditis, phlebitis, osteomyelitis, and meningitis [34]. While *E. coli* can cause urinary tract infections, new-born meningitis, pneumonia, traveller’s diarrhoea, and bacteraemia, *B. subtilis* can cause food poisoning [35]. Meningitis, pneumonia, wound site or surgical site infections, and bloodstream infections are all caused by *K. pneumonia*. Diarrhoea is caused by *S. dysenteriae*, a gram-negative bacterium. Infections of the urinary tract can be caused by the gram-negative bacterium *P. vulgaris* [36,37]. Additionally, they create urease, which raises the incidence of UTIs in the upper urinary tract. Infections of the skin, soft tissues, skeleton, and eyes can all be caused by the gram-negative bacterium *P. aeruginosa*, which can also cause a wide range of other illnesses [38].

For the first-time evaluations of *N. procumbens* fractions against *S. aureus, B. subtilis, E. coli, K. pneumonia, S. dysenteriae, P. vulgaris*, and *P. aeruginosa* were conducted. All fractions of *N. procumbens* exhibited moderate antibacterial activity, with zones of inhibition ranging from 7 to 19 mm against several harmful bacteria. The maximum zone of inhibition measured against *K. pneumoniae* was 19 mm for the *n*-butanol fraction. The crude extract fraction with the lowest efficacy against P. vulgaris was 7 mm. In the current study, methanolic extract of *N. procumbens* showed a zone of inhibition ranging from 7 to 16 mm against various bacteria, *n*-hexane fraction showed a zone of inhibition ranging from 9 to 17 mm, chloroform fraction showed a zone of inhibition ranging from 12 to 19 mm, and *n*-butanol fraction also showed a zone of inhibition ranging from 12 to 19 mm against various bacteria. Methanol extract showed the largest zone of inhibition against *E. coli*; *n*-hexane fraction against *P. aeruginosa*; chloroform fraction against *S. aureus* and *E. coli*; and *n*-butane fraction against *K. pneumoniae*. According to the findings of the present investigation, the fractions were fairly efficient against both gram-positive and gram-negative bacteria.

In previous studies, β -sitosterol, stigmasterol, phytol, and ferulic acid were isolated and identified from *N. procumbens*, and phytochemical analysis revealed the presence of flavonoids [39,40]. Phytol has also been reported to be antibacterial against *P. aeruginosa* [41]. Antibacterial activity of flavonoids has also been reported [42]. In addition to antibacterial activity against *S. aureus, E. coli*, and *P. aeruginosa*, the mechanism of action for ferulic acid’s antibacterial activity has been studied [43]. Thus, it can be concluded that the presence of all these compounds in *N. procumbens* is collectively responsible for its antibacterial activity. As *N. procumbens* showed zones of inhibition against both gram-positive and gram-negative bacteria, this herb has the potential to cure diseases of the respiratory, digestive, urinary, and bloodstreams as well as of the skin and soft tissues.

Molecular docking was performed to theoretically examine ligand-enzyme interactions in order to comprehend the molecular foundation for the many biological functions of natural products. It offers an improved understanding of the unique mode of action and binding affinity of active ligands against enzymes. Computational molecular docking has become the norm in the pharmaceutical industry nowadays. Scientists can use docking to identify protein binding complexes and the type of interactions between research substances at the enzyme or receptor site. Molecular docking was performed on the xanthine oxidase enzyme. There are several ligand docking simulations in the protein binding sites of PyRx, which makes virtual screening possible [44,45]. A total of 26 ligands were docked with the enzymes. Hydrogen bonds play a critical role in protein-ligand interactions and hydrophobic interactions, such as alkyl and pi alkyl, as well as the long-term binding of ligands to proteins [46]. Findings from molecular docking show how the xanthine oxidase enzyme interact with the ligands caryophyllene, β-amyrin, stigmasterol, ergosta-4,6,22-trien-3-ol, and campesterol validating our in vitro enzyme inhibition results.

## 4. Materials and Methods

### 4.1. Apparatus

Digital rotary evaporator (Heidolph Laboratory, Schwabach, Germany), microplate reader (Synergy HT BioTek, Santa Clara, CA, USA), pH metre (WTW series, Inolab, Washington, DC, USA) and digital weighing balance (Uni Bloc, Shimadzu, AUW220D, Gujarat, India).

### 4.2. Plant Collection and Extraction

The mature plant was collected in May 2016 from the Cholistan Desert in the Bahawalpur area of Pakistan. Taxonomist from the Department of biological sciences at The Islamia University of Bahawalpur, Pakistan, validated and assigned voucher number 452/LS to the identified plant. The plant was grinded into a coarse powder before being immersed for 15 days in 80 percent methanol. Initially, muslin cloth was used for filtration, followed by filter paper (Whatman No. 1). The filtrate was then condensed using a rotary evaporator at 40 °C and lowered pressure. It was then divided using organic solvents based on polarity, i.e., *n*-hexane, chloroform, and *n*-butanol, and the leftover aqueous fraction was also employed.

### 4.3. Phytochemical Analysis

#### 4.3.1. Preliminary Qualitative Phytochemical Screening

In accordance with the standard procedures described in [47,48], preliminary qualitative phytochemical screening was performed on *N. procumbens* methanol extract and various fractions for the detection of carbohydrates, glycosides, flavonoids, proteins and amino acids, steroids and tannins as well as for the detection of alkaloids and resins, quinones and lipids.

#### 4.3.2. HPLC-PDA Analysis

Using a Waters 600 solvent pump and 2996 photodiode array detector, the HPLC analysis was carried out using Empower v.2 software (Waters Spa, Milford, MA, USA) for data acquisition. A Jetstream2 Plus column oven was used to heat the C18 reversed-phase packing column to 30 ± 1 °C (Prodigy ODS (3), 4.6 150 mm; 5 m; Phenomenex, Torrance, CA, USA). Set to a range of 200–500-nanometres, the UV/Vis acquisition wavelength each compound’s maximum wavelength was used for the quantitative analysis. The injection volume was 20-microliters. Biotech DEGASi, mod. Compact (LabService, Anzola dell’Emi-lia, Italy) was used to degas the mobile phase directly on-line. The gradient elution was carried out as described in the literature, using the mobile phase water-acetonitrile (93:7) with 3 percent acetic acid [49]. HPLC was used to separate the resulting supernatant from all prepared sample solutions.

#### 4.3.3. GC-MS Analysis

Sample volume of 1-microliter was injected using a hot-needle approach in split-less mode for GC-MS analysis. There were three major components to the system: the automated liquid sampler (ALS), the gas chromatograph, and the mass spectrometer (Agilent, Santa Clara, CA, USA). Following an integrated guard column, HP-5 MS capillary columns of 0.25 mm inner diameter and 0.25 m film were utilised for Gas Chromatography integration’s (Agilent, Santa Clara, CA, USA). Ion source and interface temperatures were both regulated to 250 °C, and injection was set to 230 °C. One millilitre per minute (mL/min) of helium was used as a carrier gas. Two minutes of 60 °C isothermal heating was followed by a 5-degree-minute^−1^ oven temperature ramp to 80 °C and a final 5-min heating at 10 °C–310 °C in the temperature protocol. Prior to injecting the next sample, the temperature was equilibrated for 6 min at 70 °C. A scanning range of 50–650 *m*/*z* was used for the mass spectra. Agilent MSD ChemStation software was used to analyse spectra and chromatograms. Corrective actions were taken to address any errors that had been made in the previously processed data [50].

### 4.4. Biological Screening

#### 4.4.1. Antioxidant Activity

By employing a stable free radical 2,2-diphenyl-1-picryl-hydrazyl (DPPH), the antioxidative activity of crude extract and all four fractions was tested. 1 DPPH in methanol solution of 0.1 mM was employed. The extract/fraction solution in methanol and the 0.1 mM DPPH solution were applied to each well of a 96-well microplate in the amounts of 10 μL and 90 μL, respectively. It was conducted twice for each experiment. For 30 min, the microplate was incubated at 37 degrees Celsius. A BioTek Synergy Microplate reader set to 517 nm measured the absorbance. The IC_50_ for DPPH inhibition was determined by making series of dilutions of stock solution [51].

#### 4.4.2. Enzyme Inhibition Assays

- **Xanthine Oxidase**

A previously described technique was conducted for oxidase inhibition assay [22]. We used a 50 mM potassium phosphate buffer pH 7.4 to dissolve the xanthine oxidase (0.003 units/well). 15 min at 30 °C were spent incubating each well with 140 μL of buffer, 20 μL of extract solution, and 20 μL of XO solution. At 295nm, the pre-read was taken. For 30 min after the pre-reading, 0.15 mM xanthine (20 μL) was added and re-incubated. After that, a 295 nm read was made. The study’s positive control was the drug allopurinol, which was used as the primary treatment in the experiment. In order to calculate the IC_50_, serial dilutions were subjected to XO inhibition testing [52].

% Inhibition = 100 − [(OD of after read − OD of pre read)/OD of control] × 100

- **Carbonic Anhydrase**

The method previously published for the carbonic anhydrase inhibition experiment was modified [53]. Somewhat. DMSO extract solution in Tris HEPES buffer of pH 7.4 was added to a well containing 140 μL of Tris HEPES buffer of pH 7.4. Pre-read was taken at 400 nm and 20 μL of 4-nitrophenol acetate in ethanol (0.7 mM) as a substrate was added for 30 min and re-incubated. After that, a 400 nm read was taken. Positive control acetazolamide was employed in the study. % Inhibition was computed as follows:

% Inhibition = 100 − [(OD of after read − OD of pre read)/OD of control] × 100

- **Urease**

To execute the anti-urease experiment, the approach was slightly altered [54]. The indo phenol method was used to measure ammonia generation as a sign of urease activity. After 15 min of incubation at 37 °C, the first test chemical, 15 μL (0.5 mg/mL), 20 μL phosphate buffer, and 15 μL of enzyme were added to 96-well plates. Substrate (urea) was then added and re-incubated at the same temperature and conditions. After incubation, the absorbance was measured at a wavelength of 630 nm. Pre-read figures were used to document the data. For the next 50 min, the mixture was incubated with 45 μL of phenol (and 70 μL of alkali) reagent. After incubation, the absorbance was measured at 630 nm and taken as a reading. Positive control thiourea and control ethanol were utilized. Percent Inhibition was calculated using the equation below:

% inhibition = 100 − (Absorbance of post read − Absorbance of pre read/Absorbance of control)

#### 4.4.3. Antibacterial Activity

The antimicrobial test was adjusted as detailed in already reported method [55]. Bacterial culture was prepared to a density of 108 cells mL^−1^ of 0.5 McFarland standard. To make a stock solution of 5 mg/mL, DMSO and crude extract were mixed together. Agar (Mueller-Hinton) was placed into petri dishes and allowed to harden for 20 min. A sterile cotton tip swab was used to evenly distribute 60 μL of bacterial solution on solidified agar. Wells were drilled into the agar surface using a cork borer and filled with 20 μL of extract solution. At this point, the petri dishes had been incubated for 24 h at 37 degrees Celsius. Using ciprofloxacin as a reference, the antibacterial activity of the extract solutions was determined by measuring the zones of inhibition in triplicate.

### 4.5. In Silico Studies

In computer-aided drug design studies, molecular docking is a particularly useful technique. At least 3 A° resolution is required for the PDB-formatted PDB of xanthine oxidase, which was retrieved from the Protein Data Bank (PDB). Discovery Studio 2021 Client was used for protein processing. Protein molecules were stripped of all save the A chain, hetatoms, water molecules, and active ligands. Proteins were then given polar hydrogen molecules and stored in a Protein Data Bank file. It was acquired from PubChem’s database in the SDF format to save the secondary metabolites from gas chromatography-mass spectrometry (GC-MS). Preparation of protein molecules for autodocking in PyRx programme has now been completed. In PyRx, the ligands from Open Babel were reduced in energy. PDBQT format was then used to convert the ligands. It was then necessary to design a unique grid box using those measurements. Interactions were ultimately visualized using Discovery studio [56].

### 4.6. Statistical Analysis

There were three replicates of each experiment, and the data was given as an average standard deviation (standard deviation). An ANOVA and Tukey’s test were used to compare the means. SPSS statistic version 17.0 was used to analyse the data and a *p*-value of 0.05 or less was considered statistically significant.

## 5. Conclusions

The present study compares the biological characteristics and chemical characterization of various solvent extracts from the plant *N. procumbens*. Preliminary phytochemical studies and HPLC-PDA analysis predicted that the methanol, chloroform, and *n*-butanol extracts would have excellent antioxidant properties due to high levels of phenolic and flavonoid contents. Analytical results from GC-MS analysis of *n*-hexane fraction showed that phenolic, sesquiterpenoids & steroids were the most prominent classes. All of the extracts examined had varying inhibitory potentials against the enzymes that were being studied. A variety of ligands were docked in their respective complexes to analyse and compare the critical molecular interactions that arise. According to the findings, *N. procumbens* is a biologically active plant with antioxidant, anti-epilepsy, anti-uricemic, anti-ulcer, and antibacterial characteristics. In order to obtain the complete picture, more research on the drug’s toxicity and bioavailability is required.

## Figures and Tables

**Table 1:** Preliminary phytochemical screening of *Neurada procumbens* tested extract/fractions.

Metabolites	Phytochemical Test	Methanol	*N*-Hexane	Chlorofom	*N*-Butanol	Aqueous
**Primary Metabolites**						
Carbohydrate	Molisch’s test	+	+++	−	+++	+
Amino Acid	Ninhydrin	−	−	−	−	−
Starch	Iodine test	−	−	−	−	−
Proteins	Biuret test					
**Secondary Metabolites**						
Tannins	Ferric-Chloride test	+	−	++	+++	+
Flavonoids	Amyl Alcohol test	+	+++	+	+++	+
Soponins	Frothing test	+	−	+++	−	+
Steroids/Terpenes	Salkowski reaction	+	++	+	++	+
Resins	Aceticanhydride test	+	++	+	−	+
Phenols	Lead acetate test	+	++	+++	++	+

Negative sign (−) indicates absence while Positive sign (+) = low concentration; ++ = moderate concentration; +++ = high concentration. All of the procedures were carried out thrice.

**Table 2:** HPLC-PDA polyphenolic composition of *N. procumbens* (μg/g sample).

Phenolic Compounds	Fractions			
	Methanolic	Chloroform	*N*-Hexane	*N*-Butanol
Gallic acid	nd	nd	BLD	nd
Catechin	nd	nd	nd	BLD
Syringic acid	nd	nd	nd	3.6 ± 0.4 ^a^
3-OH benzoic acid	nd	1.9 ± 0.2 ^b^	nd	nd
*t*-ferullic acid	1.7 ± 0.6 ^a^	0.3 ± 0.02 ^c^	nd	nd
Naringin	nd	nd	nd	0.5 ± 0.05 ^b^
Quercetin	nd	nd	BLD	nd
Harpagoside	nd	2.4 ± 0.8 ^a^	nd	nd

Different superscript letters present statistically significant differences in the extracts for each species (*p* < 0.05) nd: not detected; BLD: below limit of detection. All of the procedures were carried out thrice.

**Table 3:** GC-MS analysis of *Neurada procumbens n*-hexane fraction.

Sr.	RT (min)	% Area	Tentative Identification	Mol. Weight	Mol. Formula	Chemical Class
**1**	9.47	0.30	Caryophyllene	204.35	C_15_H_24_	Sesquiterpenoid
**2**	14.11	1.25	Bicyclo[3.1.1]heptane, 2,6,6-tr.	138.25	C_10_H_18_	Terpene
**3**	14.36	0.20	5-Nonadecen-1-ol	282.5	C_19_H_38_O	Alcohol
**4**	15.26	2.56	*n*-Hexadecanoic acid	256.4	C_16_H_32_O_2_	Fatty acid
**5**	17.84	1.33	9,12,15-Octadecatrienoic acid	278.4	C_18_H_32_O_2_	Fatty acid
**6**	17.13	2.47	Phytol	296.5	C_20_H_40_O	Diterpenoid
**7**	17.46	0.83	Octadecanoic acid	298.5	C_19_H_38_O_2_	Fatty acid
**8**	20.89	0.36	Eicosanoic acid, methyl ester	326.6	C_21_H_42_O_2_	Fatty acid
**9**	23.33	0.88	Docosanoic acid	354.6	C_23_H_46_O_2_	Fatty acid
**10**	23.83	1.76	1,2-Benzenedicarboxylic acid	166.1	C_8_H_6_O_4_	Carboxylic acid
**11**	26.64	0.43	Tetracosanoic acid	382.7	C_25_H_50_O_2_	Fatty acid
**12**	29.22	0.89	Methoxyacetic acid	328.5	C_20_H_40_O_3_	Carboxylic acid
**13**	29.76	6.37	Cholest-5-en-3-ol (3β)	386.7	C_27_H_46_O	Cholesterol
**14**	30.56	12.45	Stigmastan-3,5,22-trien	394.7	C_29_H_46_	Steroid
**15**	30.23	2.88	(22*E*)-Ergosta-4,6,22-trien-3-ol	396.6	C_28_H_44_O	Steroid
**16**	31.89	19.42	Stigmastan-3,5-diene	396.7	C_29_H_48_	Steroid
**17**	31.44	0.16	1-(3-Hydroxy-4-methylphenyl)-	296.4	C_2o_H_24_O_2_	Phenol
**18**	32.26	0.22	d,α-Tocopherol	430.7	C_29_H_50_O_2_	Phenol
**19**	33.63	3.79	Campesterol	400.7	C_28_H_48_O	Sterol
**20**	35.85	4.88	Octacosane	394.8	C_28_H_58_	Alkane
**21**	35.33	6.36	Stigmasterol	412.7	C_29_H_48_O	Sterol
**22**	36.47	0.99	β-Amyrin	426.7	C_30_H_50_O	Triterpenoid

RT: retention time; Mol. formula: molecular formula; Mol. weight: molecular weight.

**Table 4:** Antioxidant activity (DPPH) of *Neurada procumbens*.

Plant Extract	DPPH	
	% Inhibition	IC_50_ (mg/mL)
Methanolic extract	81.4 ± 0.7 ^a^	1.1 ± 0.3 ^a^
*n*-hexane fraction	67.2 ± 0.8 ^c^	2.2 ± 0.5 ^c^
Chloroform fraction	43.5 ± 0.6 ^d^	6.4 ± 1.8 ^d^
*n*-butanol fraction	74.2 ± 0.6 ^b^	1.5 ± 0.3 ^b^
Aqueous fraction	18.8 ± 0.9 ^e^	-
Ascorbic acid	93.7 ± 0.1 ^a^	0.004 ± 0.5 ^e^

Values are mean (*n* = 3) ± SD. Superscript a-f show that mean ± standard error of mean with different superscript are significantly different (*p* < 0.05). Ascorbic acid 93.74% inhibition at 0.5 mmol. All of the procedures were carried out thrice. (–) = “Negligible”

**Table 5:** Enzyme inhibition assays of *Neurada procumbens* extract/fractions.

Extract/Fractions	Enzyme Inhibition					
	Xanthine Oxidase		Carbonic Anhydrase		Urease	
	% Age Inhibition	IC_50_ (mg/mL)	% Age Inhibition	IC_50_ (mg/mL)	% Age Inhibition	IC_50_ (mg/mL)
Methanolic extract	58.3 ± 0.4 ^c^	3.9 ± 0.2	78.4 ± 0.2 ^b^	2.2 ± 0.4	54.2 ± 1.7 ^d^	3.7 ± 0.7 ^d^
*n*-hexane fraction	79.5 ± 0.9 ^b^	2.3 ± 0.5	32.7 ± 0.3 ^c^	-	84.7 ± 1.9 ^a^	1.1 ± 0.4 ^a^
Chloroform fraction	56.3 ± 0.4 ^c^	4.1 ± 0.3	17.6 ± 0.2 ^e^	-	81.6 ± 1.2 ^b^	1.8 ± 0.8 ^b^
*n*-butanol fraction	24.8 ± 0.8 ^e^	-	67.9 ± 0.6 ^c^	3.1 ± 0.4	67.5 ± 1.2 ^c^	2.4 ± 0.3 ^c^
Aqueous fraction	39.7 ± 0.3 ^d^	-	24.8 ± 0.1 ^d^	-	42.7 ± 1.9 ^e^	-
Allopurinol	92.0 ± 0.2 ^a^	0.04 ± 0.4	-	-	-	-
Acetazolamide	-	-	98.1 ± 0.02 ^a^	0.03 ± 0.3	-	-
Thio-urea	-	-	-	-	94.3 ± 0.5 ^a^	0.14 ± 0.3 ^a^

All of the procedures were carried out thrice. Values articulated as mean (*n* = 3) ± standard deviation. Statistical analysis was conducted using ANOVA. Significantly different results were exhibited when compared to standard (*p* < 0.05). (–) = “Negligible”.

**Table 6:** Antibacterial activity of *Neurada procumbens* tested extract/fractions.

	Zone of Inhibition (mm) Mean ± SEM						
	*S. aureus*	*S. dysenteriae*	*E. coli*	*P. aeruginosa*	*B. subtilis*	*P. vulgaris*	*K. pneumoniae*
Methanol	12 ± 0.1 ^b^	13 ± 0.3 ^b^	16 ± 0.8 ^b^	14 ± 0.3 ^b^	10 ± 0.4 ^c^	7 ± 0.2 ^c^	8 ± 0.7 ^c^
*N*-Hexane	13 ± 0.3 ^b^	15 ± 0.6 ^a^	12 ± 0.4 ^c^	17 ± 0.6 ^a^	13 ± 0.2 ^b^	9 ± 0.7 ^c^	11 ± 0.3 ^c^
Chloroform	15 ± 0.5 ^a^	13 ± 0.2 ^b^	19 ± 0.1 ^a^	13 ± 0.2 ^b^	12 ± 0.6 ^b^	13 ± 0.4 ^b^	13 ± 0.9 ^b^
*N*-Butanol	12 ± 0.1 ^b^	15 ± 0.4 ^a^	12 ± 0.5 ^c^	12 ± 0.4 ^b^	14 ±0.3 ^a^	12 ± 0.2 ^b^	19 ± 0.1 ^a^
Aqueous	8 ± 0.3 ^c^	2 ± 0.2 ^c^	-	6 ± 0.1 ^c^	-	5 ± 0.4 ^c^	7 ± 0.6 ^c^
Ciprofloxacin	30 ± 0.3	34 ± 0.1	27 ± 0.3	34 ± 0.1	27 ± 0.2	30 ± 0.5	32 ± 0.3

All of the procedures were carried out thrice. Values articulated as mean (*n* = 3) ± standard deviation. Statistical analysis was conducted using ANOVA. Significantly different results were exhibited when compared to standard (*p* <0.05). (–) = “Negligible”.

**Table 7:** Binding affinities and interactions of the selected phytocompounds identified by GC-MS from *N. procumbens n*-hexane fraction against xanthine oxidase enzyme.

Enzyme	Ligand	Binding Affinity (Kcal/mol)	Amino Acids Interactions
Xanthine oxidase	β-Amyrin	−9.7	Conventional Hydrogen Bond: (LEU^A36^ GLU^A89^) Van de Waals: (GLY^A35^, ARG^A37^, GLY^A38^, VAL^A88^, ALY^A92^, SER^A93^, THR^A94^, LYS^A95^, LEU^A98^, GLN^A102^, THR^A117^, PRO^A118^, VAL^A121^)
	Campesterol	−7.6	Alkyl: (VAL^A88^, ALA^A106^) Van der Waals: (GLY^A38^, GLU^A89^, GLY^A92^, SER^A93^, THR^A94^, LYS^A95^, LEU^A98^, GLN^A102^, ILE^A105^, SER^A111^, GLY^A114^, THR^A117^, PRO^A118^, VAL^A121^)
	Ergosta-4,6,22-trien-3-ol	−7.6	Alkyl: (VAL^A88^, LEU^A98^, PRO^A118^, ALA^A106^, PRO^A118^) Van de Waals: (GLY^A35^, LEU^A36^, ARG^A37^, GLY^A38^, GLU^A89^, SER^A93^, THR^A94^, LYS^A95^, GLN^A102^, ILE^A105^, SER^A111^, THR^A117^, VAL^A121^)
	Stigmasterol	−7.5	Conventional Hydrogen Bond: (ARG^A37^) Van de Waals: (GLY^A35^, LEU^A36^, GLY^A38^, VAL^A88^, GLU^A89^, SER^A93^, THR^A94^, LYS^A95^, LEU^A98^, GLN^A102^, ILE^A105^, ALA^A106^, THR^A117^, PRO^A118^, VAL^A121^)
	Caryophyllene	−6.3	Alkyl: (VAL^A88^, PRO^A118^) Van de Waals: (GLU^A89^, GLY^A92^, SER^A93^, THR^A94^, LEU^A98^, GLN^A102^, THR^A117^, VAL^A121^)
	Allopurinol (standard)	−5.2	Unfavourable Donor: (THR^A52^) Van der Waals: (GLY^A49^, SER^A69^, SER^A123^) Conventional Hydrogen Bond: (ASN^A71^, ALA^A142^, GLY^A145^, ASN^A146^)

## Data Availability

Not applicable.

## Sample Availability

Samples of the compounds are available from the authors.

## References

[1] Toiu A., Mocan A., Vlase L., Pârvu A.E., Vodnar D.C., Gheldiu A.M., Moldovan C., Oniga I. (2018). Phytochemical composition, antioxidant, antimicrobial and in vivo anti-inflammatory activity of traditionally used Romanian Ajuga laxmannii (Murray) Benth.(“Nobleman’s Beard”–Barba Împăratului). Front. Pharmacol..

[2] Baessa M.P., Rodrigues M.J., Pereira C.G., Santos T.F., da Rosa Neng N., Nogueira J.F., Barreira L., Varela J.C., Ahmed H.M., Asif S. (2019). A comparative study of the in vitro enzyme inhibitory and antioxidant activities of *Butea monosperma* (Lam.) Taub. and *Sesbania grandiflora* (L.) Poiret from Pakistan: New sources of natural products for public health problems. S. Afr. J. Bot..

[3] Koehn F.E., Carter G.T. (2005). The evolving role of natural products in drug discovery. Nat. Rev. Drug Discov..

[4] Pierangeli G.V., Windell L.R. (2009). Antimicrobial activity and cytotoxicity of *Chromolaena odorata* (L. f.) King and Robinson and *Uncaria perrottetii* (A. Rich) Merr. Extracts. J. Med. Plants Res..

[5] Cvetanović A., Zeković Z., Švarc-Gajić J., Razić S., Damjanović A., Zengin G., Delerue-Matos C., Moreira M. (2018). A new source for developing multi-functional products: Biological and chemical perspectives on subcritical water extracts of *Sambucus ebulus* L.. J. Chem. Technol. Biotechnol..

[6] Farnsworth N.R., Bingel A.S. (1977). Natural Products and Plant Drugs with Pharmacological, Biological or Therapeutic Activity.

[7] Fisher P.R. (1991). The role of gaseous metabolites in phototaxis by *Dictyostelium discoideum* slugs. FEMS Microbiol. Lett..

[8] Verma S., Singh S. (2008). Current and future status of herbal medicines. Vet. World.

[9] Jachak S.M., Saklani A. (2007). Challenges and opportunities in drug discovery from plants. Curr. Sci..

[10] Marwat Q., Siddiqui M. *Neurada procumbens* L. Pakistan Plant Database. http://www.tropicos.org/Name/27802186.

[11] Malik S., Ahmad S., Sadiq A., Alam K., Wariss H.M., Ahmad I., Hayat M.Q., Anjum S., Mukhtar M. (2015). A comparative ethno-botanical study of Cholistan (an arid area) and Pothwar (a semi-arid area) of Pakistan for traditional medicines. J. Ethnobiol. Ethnomed..

[12] Marzouk M.M., Hussein S., Ibrahim L.F., Elkhateeb A. (2014). Flavonoids from *Neurada procumbens* L. (Neuradaceae) in Egypt. Biochem. Syst. Ecol..

[13] Ali M.A., Abul Farah M., Al-Hemaid F.M., Abou-Tarboush F.M. (2014). In vitro cytotoxicity screening of wild plant extracts from Saudi Arabia on human breast adenocarcinoma cells. Genet. Mol. Res..

[14] Shahzad M.I., Anwar S., Ashraf H., Manzoor A., Naseer M., Rani U., Aslam Z., Saba N., Kamran Z., Ali S. (2019). Study of antiviral potential of cholistani plants against new castle disease virus. Pak. J. Zool..

[15] Chen H.B., Islam M.W., Radhakrishnan R., Wahab S.A., Naji M.A. (2004). Influence of aqueous extract from *Neurada procumbens* L. on blood pressure of rats. J. Ethnopharmacol..

[16] Kapoor B., Kumar S. (2013). Ethnomedicinal plants of Barmer District, rajasthan used in herbal and folk remedies. Indian J. Pharm. Biol. Res..

[17] Qureshi R., Bhatti G.R., Memon R.A. (2010). Ethnomedicinal uses of herbs from northern part of Nara desert, Pakistan. Pak J Bot..

[18] Moustafa S.M., Menshawi B.M., Wassel G.M., Mahmoud K. (2014). Screening of some plants in Egypt for their cytotoxicity against four human cancer cell lines. Int. J. PharmTech Res..

[19] Dilworth L., Riley C., Stennett D. (2017). Plant constituents: Carbohydrates, oils, resins, balsams, and plant hormones. Pharmacognosy.

[20] Singh A.P., Kumar S. (2018). Applications of tannins in industry. Tannins-Structural Properties, Biological Properties and Current Knowledge.

[21] Riaz A., Rasul A., Hussain G., Zahoor M.K., Jabeen F., Subhani Z., Younis T., Ali M., Sarfraz I., Selamoglu Z. (2018). Astragalin: A bioactive phytochemical with potential therapeutic activities. Adv. Pharmacol. Sci..

[22] Thomson R. (2012). Naturally Occurring Quinones.

[23] Riveiro M.E., De Kimpe N., Moglioni A., Vázquez R., Monczor F., Shayo C., Davio C. (2010). Coumarins: Old compounds with novel promising therapeutic perspectives. Curr. Med. Chem..

[24] Aktumsek A., Zengin G., Guler G.O., Cakmak Y.S., Duran A. (2013). Antioxidant potentials and anticholinesterase activities of methanolic and aqueous extracts of three endemic *Centaurea* L. species. Food Chem. Toxicol..

[25] Seth R., Kydd A.S., Buchbinder R., Bombardier C., Edwards C.J. (2014). Allopurinol for chronic gout. Cochrane Database Syst. Rev..

[26] Borges F., Fernandes E., Roleira F. (2002). Progress towards the discovery of xanthine oxidase inhibitors. Curr. Med. Chem..

[27] Mishra C.B., Tiwari M., Supuran C.T. (2020). Progress in the development of human carbonic anhydrase inhibitors and their pharmacological applications: Where are we today?. Med. Res. Rev..

[28] Mincione F., Scozzafava A., Supuran C.T. (2009). Antiglaucoma Carbonic Anhydrase Inhibitors as Ophthalomologic Drugs.

[29] Cherecheanu A.P., Garhofer G., Schmidl D., Werkmeister R., Schmetterer L. (2013). Ocular perfusion pressure and ocular blood flow in glaucoma. Curr. Opin. Pharmacol..

[30] Quigley H.A., Broman A.T. (2006). The number of people with glaucoma worldwide in 2010 and 2020. Br. J. Ophthalmol..

[31] Carradori S., Mollica A., De Monte C., Ganese A., Supuran C.T. (2015). Nitric oxide donors and selective carbonic anhydrase inhibitors: A dual pharmacological approach for the treatment of glaucoma, cancer and osteoporosis. Molecules.

[32] Hassan S.T., Žemlička M. (2016). Plant-derived urease inhibitors as alternative chemotherapeutic agents. Archiv. Pharm..

[33] Tariq S.A., Ahmad M.N., Obaidullah, Khan A., Choudhary M.I., Ahmad W., Ahmad M. (2011). Urease inhibitors from Indigofera gerardiana Wall. J. Enzym. Inhib. Med. Chem..

[34] Cantón R., Ruiz-Garbajosa P. (2013). Infections caused by multi-resistant Gram-positive bacteria (*Staphylococcus aureus* and *Enterococcus* spp.). Enferm. Infecc. Microbiol. Clin..

[35] Madappa T., Go C. *Escherichia coli* (*E. coli*) Infections. Medscape. https://emedicine.medscape.com/article/217485-overview.

[36] Cherla R.P., Lee S.-Y., Tesh V.L. (2003). Shiga toxins and apoptosis. FEMS Microbiol. Lett..

[37] Gómez-Guzmán A., Jiménez-Magaña S., Guerra-Rentería A.S., Gómez-Hermosillo C., Parra-Rodríguez F.J., Velázquez S., Aguilar-Uscanga B.R., Solis-Pacheco J., González-Reynoso O. (2017). Evaluation of nutrients removal (NO_3_-N, NH_3_-N and PO_4_-P) with Chlorella vulgaris, Pseudomonas putida, Bacillus cereus and a consortium of these microorganisms in the treatment of wastewater effluents. Water Sci. Technol..

[38] Sabat A.J., Pantano D., Akkerboom V., Bathoorn E., Friedrich A.W. (2021). Pseudomonas aeruginosa and *Staphylococcus aureus* virulence factors as biomarkers of infection. Biol. Chem..

[39] Pierre L.L., Moses M.N. (2015). Isolation and characterisation of stigmasterol and β-sitosterol from *Odontonema strictum* (acanthaceae). J. Innov. Pharm. Biol. Sci..

[40] Khurshid U., Ahmad S., Saleem H., Nawaz H.A. (2019). Phytochemical composition and in vitro pharmacological investigations of *Neurada procumbens* L. (Neuradaceae): A multidirectional approach for industrial products. Ind. Crops Prod..

[41] Lee W., Woo E.-R., Lee D.G. (2016). Phytol has antibacterial property by inducing oxidative stress response in Pseudomonas aeruginosa. Free. Radic. Res..

[42] Cushnie T.T., Lamb A.J. (2005). Antimicrobial activity of flavonoids. Int. J. Antimicrob. Agents.

[43] Borges A., Ferreira C., Saavedra M.J., Simões M. (2013). Antibacterial activity and mode of action of ferulic and gallic acids against pathogenic bacteria. Microb. Drug Resist..

[44] Chandak N., Kumar P., Kaushik P., Varshney P., Sharma C., Kaushik D., Jain S., Aneja K.R., Sharma P.K. (2014). Dual evaluation of some novel 2-amino-substituted coumarinylthiazoles as anti-inflammatory–antimicrobial agents and their docking studies with COX-1/COX-2 active sites. J. Enzym. Inhib. Med. Chem..

[45] Velika B., Kron I. (2012). Antioxidant properties of benzoic acid derivatives against superoxide radical. Free. Radic. Antioxid..

[46] Borges R.S., Lima E.S., Keita H., Ferreira I.M., Fernandes C.P., Cruz R.A.S., Duarte J.L., Velázquez-Moyado J., Ortiz B.L.S., Castro A.N. (2018). Anti-inflammatory and antialgic actions of a nanoemulsion of *Rosmarinus officinalis* L. essential oil and a molecular docking study of its major chemical constituents. Inflammopharmacology.

[47] Brain K., TD T. (1975). Wright-Scientechnica. Practical Evaluation of Phytopharmaceuticals.

[48] El-Olemy M.M., Al-Muhtadi F.J., Afifi A.-F.A. (1994). Experimental Phytochemistry: A Laboratory Manual.

[49] Locatelli M., Zengin G., Uysal A., Carradori S., De Luca E., Bellagamba G., Aktumsek A., Lazarova I. (2017). Multicomponent pattern and biological activities of seven *Asphodeline taxa*: Potential sources of natural-functional ingredients for bioactive formulations. J. Enzym. Inhib. Med. Chem..

[50] Falodun A., Siraj R., Choudhary M.I. (2009). GC-MS analysis of insecticidal leaf essential oil of *Pyrenacantha staudtii* Hutch and Dalz (Icacinaceae). Trop. J. Pharm. Res..

[51] Khurshid U., Ahmad S., Rehman T., Arshad M.A., Pervaiz I., Saba S. (2019). GC-MS analysis, DPPH & enzyme inhibition assays of *Trianthema triquetra* Rottl. and Willd. growing in Pakistan. Lat. Am. J. Pharm..

[52] Arshad M.A., Ahmad S., Khurshid U., Pervaiz I. (2018). Studies on the antioxidant and xanthine oxidase inhibition potential of *Heliotropium crispum*. Acta Pol. Pharm..

[53] Rehman T., Ahmad S., Abbasi W.M., Ahmad A., Bilal M., Zaman M.M., Ghauri A.O., Arshad A., Akhtar K. (2017). Evaluation of antibacterial and carbonic anhydrase inhibitory potential of methanolic extract of *Nardostachys jatamansi* (d. Don) dc rhizomes. Acta. Pol. Pharm..

[54] Aziz M., Ahmad S., Khurshid U., Saleem H. (2022). Phytochemical, pharmacological, and In-silico molecular docking studies of Strobilanthes glutinosus Nees: An unexplored source of bioactive compounds. S. Afr. J. Bot..

[55] Zeybek Z., Dogruoz N., Karagoz A. (2008). Antibacterial activity of some plant extracts. Eur. J. Biol..

[56] Tabassum S., Ahmad S., Rehman Khan K.U., Tabassum F., Khursheed A., Zaman Q.U., Bukhari N.A., Alfagham A., Hatamleh A.A., Chen Y. (2022). Phytochemical Profiling, Antioxidant, Anti-Inflammatory, Thrombolytic, Hemolytic Activity In Vitro and In Silico Potential of *Portulacaria afra*. Molecules.

